# Metallothioneins may be a potential prognostic biomarker for tumors: A Prisma-compliant meta-analysis

**DOI:** 10.1097/MD.0000000000013786

**Published:** 2018-12-28

**Authors:** Lei Wang, Fuli Xin, Nanping Lin, Yingchao Wang, Xiaolong Liu, Jingfeng Liu

**Affiliations:** Mengchao Hepatobiliary Hospital of Fujian Medical University, The United Innovation of Mengchao Hepatobiliary Technology Key Laboratory of Fujian Province, Fuzhou, Fujian, PR China.

**Keywords:** meta-analysis, metallothioneins, overall survival, prognostic

## Abstract

**Background:**

Metallothioneins (MTs) were reported to be associated with many kinds of tumors’ prognosis, although MTs expression varied greatly among tumors. To assess the prognostic value of Metallothioneins (MTs) in different kinds of tumors, comprehensive literature search was conducted to perform a meta-analysis.

**Methods:**

Eligible studies were identified by PubMed, MEDLINE, Web of Science (WOS), the Cochrane Library of Systematic Reviews, EMBASE, China National Knowledge Infrastructure (CNKI), WANFANG database and SinoMed database up to December 2017, which was designed to assess the prognostic value of MTs in different kinds of tumors. The main endpoint events were overall survival (OS) and disease-free survival (DFS). Hazard ratios (HRs) and its variance were retrieved from the original studies directly or calculated using Engauge Digitizer version 4.1. Random or fixed effects model meta-analysis was employed depending on the heterogeneity. Publication bias was evaluated by funnel plots, Begg and Egger tests.

**Results:**

A total of 22 studies were enrolled in this meta-analysis, including 2843 tumor tissues (1517 were MTs negative/low, and 1326 were MTs high). Results showed that there was significant association between MTs expression and tumors’ OS (HR = 1.60; 95%CI 1.34∼1.92, *P* < .00001). Subgroup analysis showed that high level of MTs expression was associated with prolonged OS in liver cancer (HR = 0.65, 95%CI 0.48∼0.89, *P* = .007), but it was on the contrary in the tumor of ovary (HR = 1.47, 95%CI 1.01∼2.14, *P* = .04), bladder (HR = 1.71, 95%CI 1.21∼2.42, *P* = .002), intestine (HR = 3.13, 95%CI 1.97∼4.97, *P* < .00001), kidney (HR = 3.31, 95%CI 1.61∼6.79, *P* = .001). However, there was no significant association between MTs expression and OS in breast (HR = 1.02, 95%CI 0.69∼1.51, *P* = .93).

**Conclusions:**

MTs could be taken as a potential prognostic biomarker for tumors, and uniqueness of MTs prognostic value in liver cancer deserved further study.

## 1 Introduction

Metallothioneins (MTs) are a family of small (6–7 kDa) protein consisting of 60 to 80 amino acids, and are identified as highly conserved among species discovered in 1957 by Margoshes and Vallee.^[1]^ Human MTs are divided into four main subgroups, i.e. MT I-IV, among of which, MT-I and MT-II are ubiquitously expressed, including in the liver, whereas MT-III and MT-IV are expressed mostly in brain tissue and squamous epithelial cells, respectively.^[2,3]^ MT-II is encoded by a single gene MT-IIA, while MT-I is encoded by a set of MT-I genes, such as MT-IA, MT-IB, MT-IE, MT-IF, MT-IG, MT-IH, and MT-IX, indicating significant heterogeneity of MT-I.^[4]^

Increasing evidence suggested that there existed considerable relationships between MTs expression and tumors.^[5–8]^ MTs were reported to be increased in tumor tissues such as bladder,^[9]^ gallbladder,^[10]^ head and neck,^[11]^ melanoma,^[12]^ ovary,^[13]^ and stomach,^[14]^ while they were decreased in breast,^[15]^ colorectal,^[16]^ hepatocellular,^[17]^ kidney,^[18]^ prostate,^[19]^ thyroid.^[20]^ MTs’ expression in tumor tissues were reported to be associated with tumorigenesis,^[21]^ progression,^[22]^ chemotherapy-resistant^[23]^ and prognosis.^[24]^ However, a comprehensive meta-analysis emphasized on the association of MTs expression and prognosis of all kinds of tumors has not been employed yet. Hence, the prognostic value of MTs were evaluated comprehensively in this meta-analysis.

## 2 Methods

This meta-analysis was performed according to the preferred Reporting Items for Systematic Reviews and Meta-Analyses (PRISMA).^[25]^ The informed consent of the patients and the ethical approval were not required since our research was based on the studies published previously.

### 2.1 Literature search

A comprehensive search was conducted by 2 independent researchers to clarify all the published researches on MTs clinical prognostic value. Both English electronic databases such as PubMed, MEDLINE, the Cochrane Library, Web of Knowledge and Chinese databases including WANFANG, CNKI, and SinoMed were used to search the literatures, from Nov. 1990 to Dec. 2017. Key words including

“Metallothioneins” and “Prognostic” combined with free text words such as “Cancer” and “Survival analysis” and “Clinical” and “Human” were identified in the electronic search. Manual search was conducted to ensure that all available studies were included in this meta-analysis, too.

### 2.2 Selection criteria

Inclusion criteria:

(1) relationships between MTs expression and OS in patients with tumors were assessed using a cohort or a case-control design;
(2) either MTs protein or mRNAs were detected in tumor tissue;
(3) patients were divided into 2 groups, namely, MTs positive and MTs negative or MTs high and MTs low, regardless of the cut-off values;
(4) Cox proportional hazard model and Kaplan-Meier curves were used for survival meta-analysis;
(5) full papers were extracted completely.

Exclusion criteria:

(1) in vitro or animal studies;
(2) case reports, letters, reviews and conference reports;
(3) studies based on overlapping cohorts deriving from the same center;
(4) sample size < 20.

Definition of MTs expression: negative/low MTs staining was encoded as MTs negative, MTs expression below the threshold or grading 0 to 2, and the remained was encoded as MTs high. Only the latest was extracted in case of the repeated extraction of papers from the same database.

### 2.3 Data extraction

All data were extracted and assessed by 2 independent investigators with predefined forms such as baseline characteristics and outcomes from each study. Hazard ratios (HRs) and its variance were retrieved from the original studies directly or calculated indirectly by a method that dependent on the results provided in the original studies. Kaplan-Meier curves were read using Engauge Digitizer version 4.1, which could acquire a considerably accurate HRs.^[26,27]^ In case of disagreement, a third investigator intervened for a decision.

### 2.4 Quality assessment

Cohort studies were assessed by Newcastle-Ottawa Scale (NOS),^[28]^ and studies with score more than 6 were considered as high quality.^[29]^

### 2.5 Statistical analysis

The systematic review and meta-analysis were registered at http://www.researchregistry.com and performed using RevMan Version 5.3 and Stata 14. The χ^2^ test and I^2^ statistics were used to assess the heterogeneity; *P* < .05 or I^2^ > 50% were considered as significant heterogeneity.^[30]^ HRs and 95%CIs were used to evaluate the relationship between MTs expression and OS. When the hypothesis of homogeneity was not rejected, the fixed-effects model was used to estimate the case with homogeneity, and the random-effects model was used for the cases with significant heterogeneity. Publication bias was evaluated by visually assessing the asymmetry of an inverted funnel plot, and then was supported quantitatively by Begg and Egger tests.^[29,31]^

## 3 Results

### 3.1 Search results

**Figure 1:**
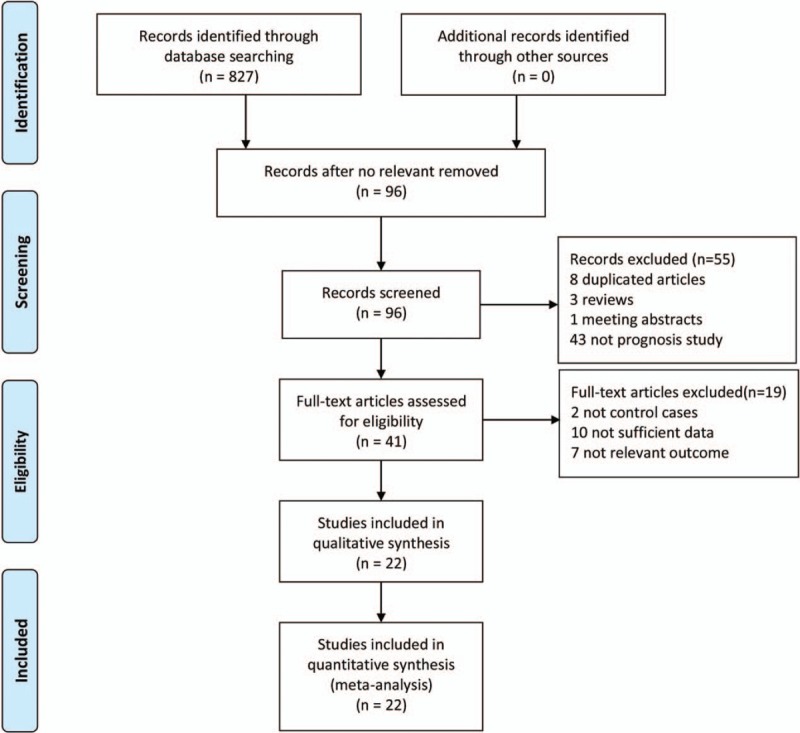
Flowchart of the study selection process for meta-analysis.

Initially, 827 reports were identified initially by 2 independent reviewers. A total of 96 articles remained after skimming through titles and abstract, and then 55 articles were excluded by preliminary screening, 8 articles were excluded after duplicate removal by NoteExpress 3.1, 3 reviews and one meeting abstract were excluded in form, and 43 articles not on prognosis were also excluded. After detailed screening, 19 articles were excluded for 2 articles without control cases, 10 articles without sufficient data, and seven articles with irrelevant outcomes (Fig. 1). Finally, 22 reports were left in this meta-analysis, including one of oral cavity,^[32]^ 1 of lung,^[33]^ 1 of lymphocyte,^[34]^ 2 of kidney,^[35,36]^ 2 of bladder,^[37,38]^ 2 of skin,^[39,40]^ 2 of colon,^[41,42]^ 3 of ovary,^[43–45]^ 3 of liver ^[46–48]^ and 5 of breast.^[49–53]^ In total, 2843 patients were enrolled in this meta-analysis, with 1517 cases in the MT negative/low group and 1326 cases in the MT high group (Table 1).

**Table 1:**
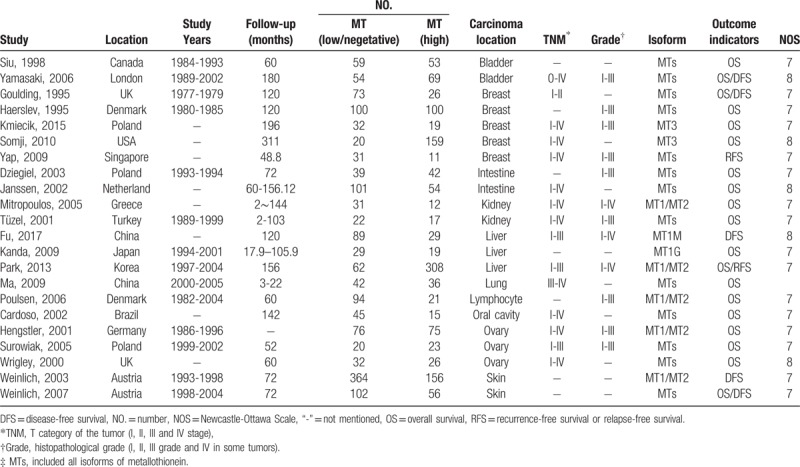
Characteristics of studies included.

### 3.2 Trial characteristics

**Figure 2:**
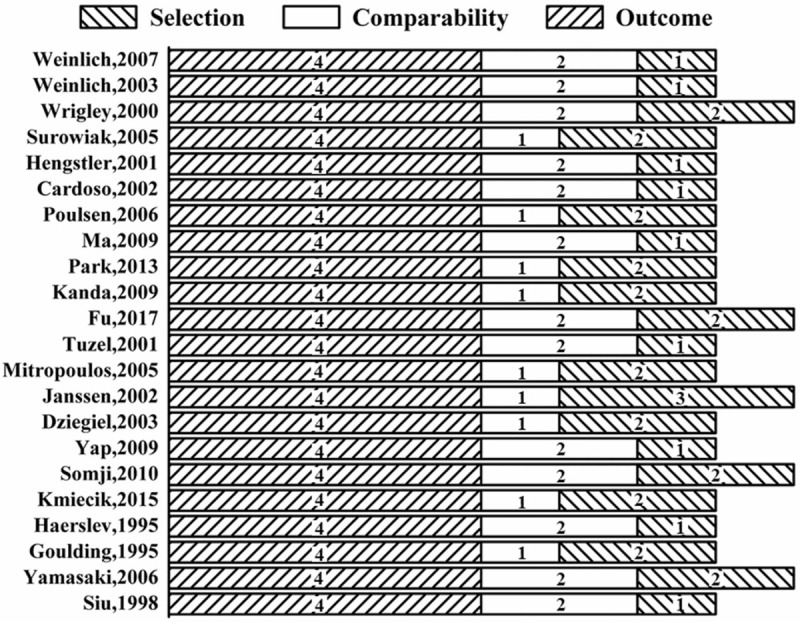
Newcastle-ottawa quality assessment scale of studies included.

The characteristics and quality of the included trials were shown in Table 1. Follow-up and the tumor feature were also mentioned in most of these studies (Table 1). All the studies including in this meta-analysis were nonrandomized studies and assessed by NOS (Fig. 2). The scores ranged from 7 to 8, indicating that all the studies were of high quality.

### 3.3 MTs could be a potential prognostic tumor biomarker in various kinds of tumors

**Figure 3:**
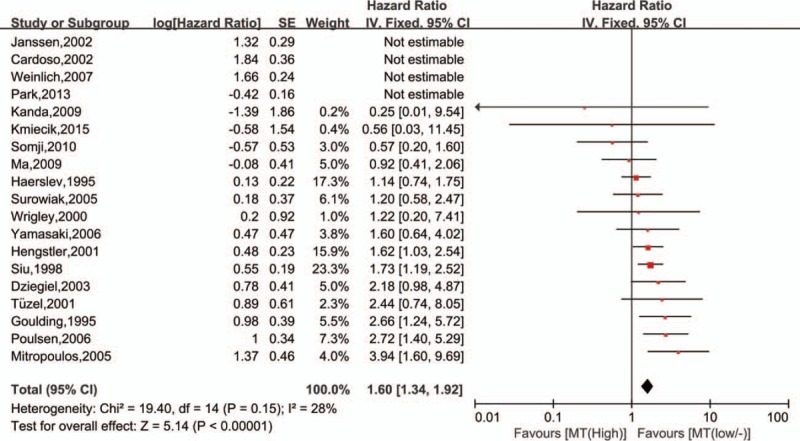
Forest plot of the association between metallothionein expression and overall survival of included studies. Not estimable meant that study was ruled out to avoid heterogeneity.

A total of 19 studies ^[32–38,40–45,47–52]^ were enrolled to evaluate the association between the OS and MTs expression, and the heterogeneity was significant among the studies (I^2^ = 82%, *P* < .00001). But, it decreased (I^2^ = 28%, *P* = .15, Fig. 3) when four studies ^[32,40,42,48]^ were excluded. Fixed-effect model was then used, and results showed that expression of MTs was significantly associated with OS (HR = 1.60; 95%CI 1.34∼1.92, *P* < .00001, Fig. 3) in tumors.

**Figure 4:**
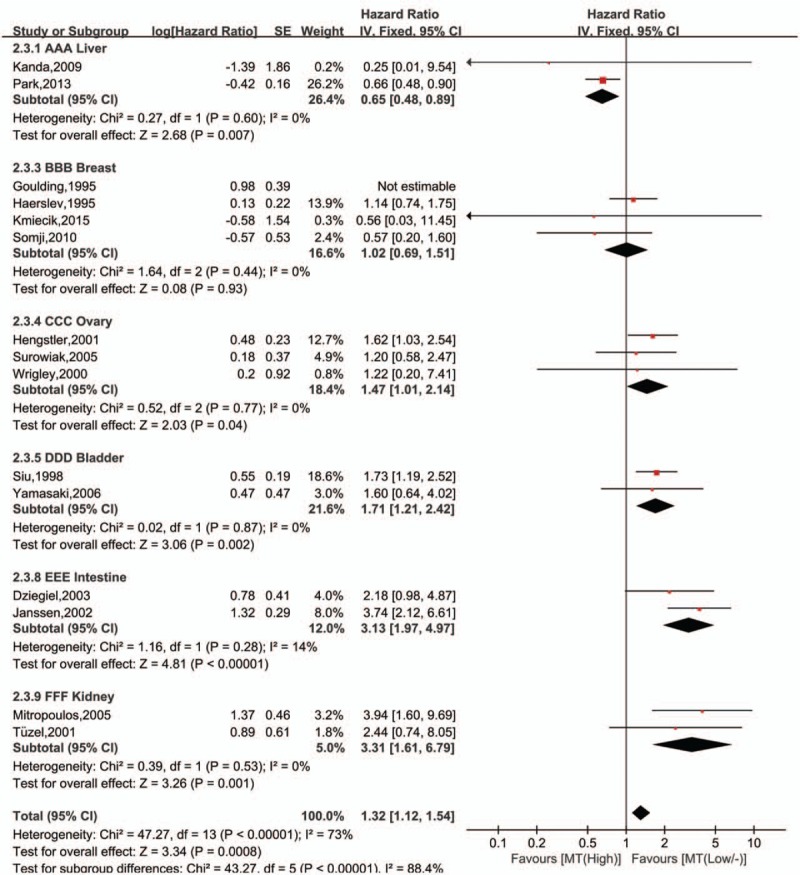
Forest plot of the association between metallothionein expression and overall survival of different organic tumors. Not estimable meant that study was ruled out to avoid heterogeneity.

Subgroup analysis was then used to eliminate the significant heterogeneity. Tumors derived from the same organ were merged to calculate a total HR (Table 2). And, only studies about liver cancer, ovary carcinoma, bladder tumor, intestine cancer, renal carcinoma, and breast cancer were furtherly analyzed in subgroup, since there was only 1 study on lung, lymphocyte, skin, and oral cavity (Fig. 4). Significant heterogeneity was found in the breast carcinoma (I^2^ = 54%, *P* = .09), but it disappeared (I^2^ = 0%, *P* = .44, Fig. 4) when one study^[49]^ was excluded. Results showed that high levels of MTs expression was associated with improved OS in liver carcinoma (I^2^ = 0%, *P* = .6; HR = 0.65, 95%CI 0.48∼0.89, *P* = .007, Fig. 4), while it was on the contrary in the tumor of ovary (I^2^ = 0%, *P* = .77; HR = 1.47, 95%CI 1.01∼2.14, *P* = .04, Fig. 4), bladder (I^2^ = 0%, *P* = .87; HR = 1.71, 95%CI 1.21∼2.42, *P* = .002, Fig. 4), intestine tumor (I^2^ = 14%, *P* = .28; HR = 3.13, 95%CI 1.97∼4.97, *P* < .00001, Fig. 4), kidney (I^2^ = 0%, *P* = .53; HR = 3.31, 95%CI 1.61∼6.79, *P* = .001, Fig. 4). However, there was no significant association between MTs expression and OS in breast cancer (I^2^ = 0%, *P* = .44; HR = 1.02, 95%CI 0.69∼1.51, *P* = .93, Fig. 4).

**Table 2:**
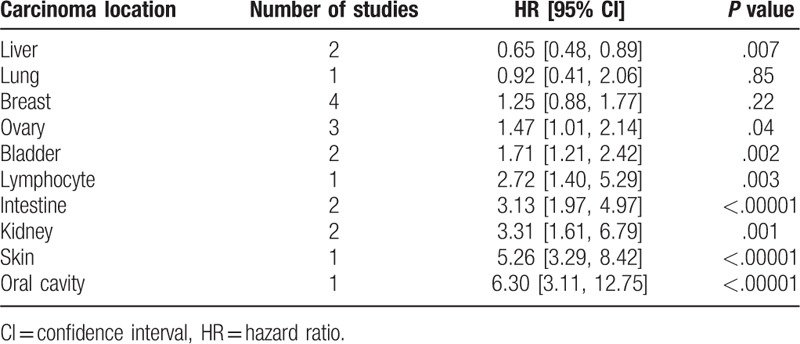
Analysis of the association between metallothionein expression and overall survival of different organic tumors.

### 3.4 Publication bias

**Figure 5:**
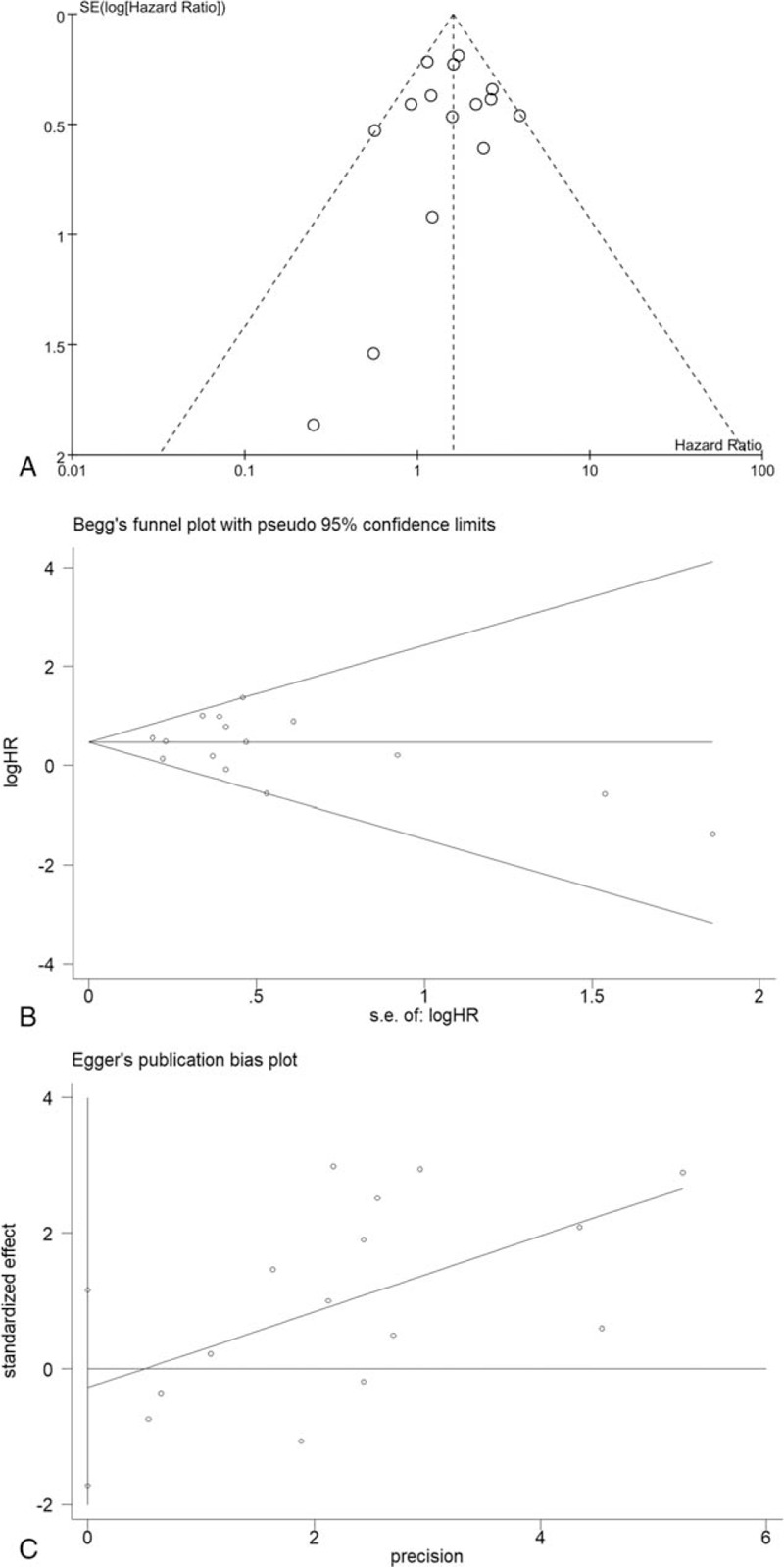
Funnel blot and Begg and Egger test exhibited publication bias. (A) Funnel plot; (B) Begg funnel plot with 95% confidence limits; (C) Egger publication bias plot.

Funnel plot and Begg and Egger tests were used to detect the publication bias of our meta-analysis. A total of 15 studies valuating the prognostic value of MTs exhibited a basically symmetrical funnel plot (Fig. 5A) and yielded a Begg (Fig. 5B) and Egger (Fig. 5C) test scores of *P* = .40 and *P* = .681 (t = -0.42, 95%CI -1.73∼1.16), respectively.

## 4 Discussion

MTs are widely expressed in various kinds of tumor cells, and were reported to be correlated with tumors’ prognosis, such as carcinoma of liver,^[17]^ ovary,^[13]^ bladder,^[9]^ intestine,^[14]^ kidney,^[18]^ melanoma,^[12]^ oral cavity.^[11]^ In this meta-analysis, MTs were confirmed to be a potential prognostic biomarker of tumors (HR = 1.60; 95%CI 1.34∼1.92, *P* < .00001), although there were significant heterogeneities among various kinds of tumors.

MTs expression varied greatly among tumors. Generally, MTs’ expression in tumor tissues was reported to be positively correlated with tumor stage, tumor grade, tumor size, metastasis, and nodal distant, while it was negatively correlated with tumor stages in kidney and stomach cancer, tumor size in colorectal cancer, tumor grade in liver cancer.^[7]^ The reasons for the difference were as follows: the expression of MTs isoforms were different even in the same kind of tumor, which might lead to various biological changes and different prognosis;^[22,54]^ On the other hand, there were significant differences among different kinds of tumors, due to tissue-specific biological characteristics.

MTs could be taken as biomarkers for tumors, but their correlations varied in different kinds of tumors. In this meta-analysis, we found that MTs overexpression was positively associated with prognosis in liver cancer (HR = 0.65, *P* = .007), which was greatly opposite to others. The mechanisms remained to be unknown, and we hypothesized that MTs’ overexpression in HCC meant a much healthier liver function, leading to a better prognosis. Since liver was the metabolism site for heavy metals, such as zinc and copper, which were highly affined to MTs.^[55]^ Besides that, MTs was reported to have a relationship with the resistance to chemotherapy,^[56]^ which meant the expression of MTs would decrease the effect of chemotherapy and lead to a worse prognostic in gastric and ovary cancers.^[44,57]^

Biomarkers, such as alpha-fetoprotein (AFP), played an important role in the diagnosis, treatment and prognosis of HCC.^[58]^ Hence, the relationship between AFP and MTs deserved further research. However, the expression of metallothionein were reported to be uncorrelated with alpha-fetoprotein (AFP) levels in Mao study (*P* = .36).^[59]^ Furthermore, AFP was reported to be uncorrelated with the expression of metallothionein both in nuclear (*P* = .258) and cytoplasm (*P* = .685) in Park study.^[48]^ And, it was reconfirmed in our current research (*P* = .054).

However, there were several limitations in this study. Firstly, the follow-up periods were greatly different from each other. Secondly, MTs expression was reported to be detected by 2 completely different immunostaining, that is, positive control or negative control,^[1]^ and the threshold varied from each other, both of which indicted an inevitable difference among studies included in this meta-analysis. Thirdly, the expression of MTs was dramatically higher at night than in the day,^[60,61]^ which meant unavoidable sampling errors. Fourthly, too few sample size in some studies ^[35,36,44,53]^ might lead a significant statistical type I error. Fifthly, all the HRs referred in the meta-analysis were calculated from survival curves, which might be less reliable than the actual HRs.^[29]^ Finally, publication bias was hardly avoided, for the journals tend to publish positive results.

Despite these drawbacks above, we could conclude that MTs could be taken as a potential prognostic tumor biomarker for tumors, indicating a promising therapeutic target in future clinical application. Interestingly, uniqueness of MTs prognostic value in liver cancer was explored in this meta-analysis, and relative work have been conducted in our laboratory, such as targeting pathway and adverse off-target effects related to MTs in liver cancer. Only a deep and comprehensive understanding of MTs and its targeted genes will make the current research come into reality.

## Author contributions

Lei Wang, Fuli Xin and Nanping Lin conception and design of the study, acquisition of data, analysis and interpretation of data, drafting the article; Yingchao Wang revising the article; Xiaolong Liu and Jingfeng Liu critical revision, final approval.

The authors declare that they have no competing interests, and all authors confirm its accuracy.

**Conceptualization:** Lei Wang.

**Investigation:** Fuli Xin, Nanping Lin.

**Methodology:** Lei Wang.

**Project administration:** Nanping Lin.

**Resources:** Fuli Xin, Nanping Lin.

**Software:** Fuli Xin, Nanping Lin.

**Supervision:** Xiaolong Liu, Jingfeng Liu.

**Validation:** Yingchao Wang.

**Visualization:** Lei Wang, Yingchao Wang, Xiaolong Liu.

**Writing – original draft:** Lei Wang.

Lei Wang orcid: 0000-0001-8975-0489.
